# The Electroretinogram I-Wave, a Component Originating in the Retinal OFF-Pathway, Associates With a Myopia Genetic Risk Polymorphism

**DOI:** 10.1167/iovs.65.13.21

**Published:** 2024-11-12

**Authors:** Zihe Xu, Jit Kai Tan, Krishnika Vetrivel, Xiaofan Jiang, Shaun M. Leo, Taha Bhatti, Ambreen Tariq, Andrew R. Webster, Anthony G. Robson, Christopher J. Hammond, Pirro G. Hysi, Omar A. Mahroo

**Affiliations:** 1 Section of Ophthalmology, King's College London, St. Thomas’ Hospital Campus, London, United Kingdom; 2 Department of Twin Research and Genetic Epidemiology, King's College London, St. Thomas’ Hospital Campus, London, United Kingdom; 3 Institute of Ophthalmology, University College London, London, United Kingdom; 4 Electrophysiology Service, Moorfields Eye Hospital NHS Foundation Trust, London, United Kingdom; 5 Genetics Service, Moorfields Eye Hospital NHS Foundation Trust, London, United Kingdom; 6 Sørlandet Sykehus Arendal, Arendal Hospital, Norway; 7 Department of Physiology, Development and Neuroscience, University of Cambridge, Cambridge, United Kingdom

**Keywords:** retina, myopia, OFF signaling pathways, electroretinography (ERG), gap junctions, retinal cone photoreceptor cells

## Abstract

**Purpose:**

One of the strongest genetic associations with myopia is near the *GJD2* gene. Recently, this locus was associated with cone-driven electroretinograms (ERGs), with findings highlighting OFF pathway signals specifically. The ERG i-wave is thought to originate in retinal OFF pathways. We explored this component and tested the hypothesis that it would be associated with the myopia risk locus.

**Methods:**

International standard LA3 ERGs, recorded with conductive fiber electrodes, were analyzed, first from patients with rare monogenic deficits impairing the ON pathway, or both ON and OFF pathways, to explore effects on the i-wave. Responses were then analyzed from adult participants from the TwinsUK cohort: i-wave amplitudes were measured by two investigators independently, blinded to genotype at the *GJD2* locus. We investigated the association between i-wave amplitude and allelic identity at this locus, adjusting for age, sex, and familial relatedness.

**Results:**

Patient recordings showed the i-wave persisted in the absence of ON pathway signals, but was abolished when both ON and OFF pathways were impaired. For TwinsUK participants, recordings and genotypes were available in 184 individuals (95% female participants; mean standard deviation [SD] age, 64.1 [9.7] years). Mean (SD) i-wave amplitude was 14.5 (SD = 6.5) microvolts. Allelic dosage at the risk locus was significantly associated with i-wave amplitude (*P* = 0.027).

**Conclusions:**

Patient ERGs were consistent with the i-wave arising from cone-driven OFF pathways. Amplitudes associated significantly with allelic dosage at the myopia risk locus, supporting the importance of cone-driven signaling in myopia development and further highlighting relevance of the OFF pathway in relation to this locus.

Rod and cone photoreceptors hyperpolarize in response to light, leading to a reduction in glutamate release at the photoreceptor to bipolar cell synapse. Rods synapse with ON bipolar cells, which depolarize in response to the reduction in glutamate release (i.e. there is a sign inversion at the synapse, whereby the postsynaptic neuron depolarizes in response to the hyperpolarization of the presynaptic neuron). Cones, however, synapse with both ON and OFF bipolar cells, which depolarize and hyperpolarize, respectively, in response to the hyperpolarization (and reduced glutamate release) at the cone synapse. Thus, parallel “ON” and “OFF” pathways are set up^1^ and these are a key feature of retinal signaling, particularly in photopic levels, reflecting depolarization or hyperpolarization, respectively, elicited by the appearance of light in the relevant part of the receptive field.

Retinal signaling is important in the process of emmetropization. Studies in chicks have shown that rates of eye growth change to compensate for lenses that have been fixed in front of the eye.^2^ That this can occur even if the optic nerve is severed indicates that a local mechanism exists whereby defocus and its direction can be detected and signaled by the retina to influence scleral growth.^2^ The importance of light signaling by the retina in refractive error development appears to be confirmed by the many genetic myopic risk loci, identified from genome wide association studies (GWAS), that are in the vicinity of genes expressed in the retina.^3^^–^^6^ In addition, several rare monogenic retinal diseases appear to also entail degrees of refractive error, with significantly shorter or longer eyes.^7^^,^^8^

Of the common genetic myopia-associated variants identified from GWAS analyses, the rs524952 locus on chromosome 15, near the *GJD2* gene, consistently shows one of the strongest associations.^3^^–^^6^ *GJD2* encodes the connexin-36 protein, which forms gap junctions, enabling electrical coupling between retinal neurons. We recently found this locus to be associated with retinal cone-driven responses, as detected in the human full-field flash electroretinogram (ERG), recorded from more than 180 adults from the TwinsUK cohort, for whom we had genotypes at the myopia risk locus.^9^ Several avenues of investigation in that study were suggestive of a specific association with cone-driven OFF bipolar cell signals.^9^

In that study, we focused on the conventionally measured flash ERG components, namely the a-wave and b-wave (Fig. 1). In the light-adapted flash ERG, there is a later positive-going component that is less well-characterized and not typically measured. The component was visible in responses published by Cobb and Morton as early as 1952.^10^ Nagata, in 1962, investigated this component in more detail, naming it the “i-wave” (to designate “interference” from some components), and suggested that it related to retinal OFF responses.^11^ In experiments some decades later in macaques, Rangaswamy et al. injected pharmacological agents intravitreally to selectively block particular inner retinal signaling pathways and concluded that the i-wave has origins in the OFF pathway, distal to (i.e. earlier in the visual signaling pathway than) the retinal ganglion cells.^12^ Figure 1 demonstrates the i-wave component, as seen in the response elicited by the international standard light-adapted flash stimulus (LA3). The troughs preceding and following the i-wave are part of the photopic negative response, reflecting signals from retinal ganglion cells.^13^^,^^14^

**Figure 1.**
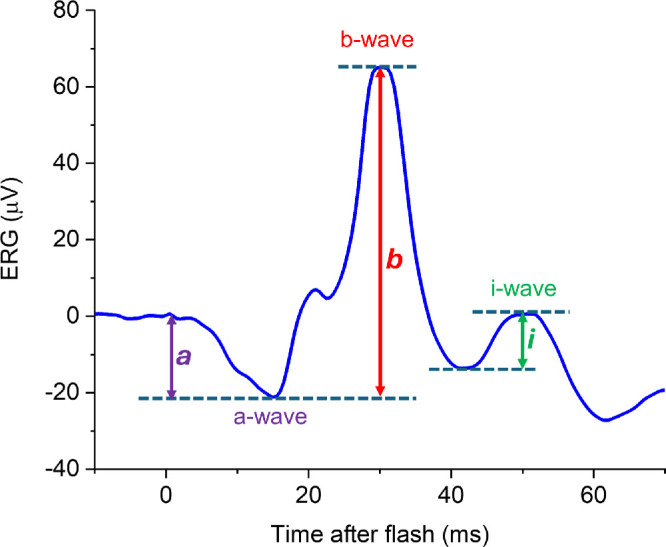
**Example ERG elicited by the international standard light-adapted single flash (LA3 ERG).** The stimulus comprises a white flash of 3 cd·s·m^−2^ delivered on a 30 cd·m^−2^ white light-adapting background through a dilated pupil (following at least 10 minutes of adaptation to the background). The trace shown is the averaged response to > 50 flashes. The conventionally measured components (a-wave and b-wave) are labeled. The a-wave amplitude is measured from the baseline to the a-wave trough (*purple double-headed arrow*); the b-wave amplitude is measured from the a-wave trough to the b-wave peak (*red double-headed arrow*). After the b-wave is a negative trough, followed by a small positive-going peak, designated the “i-wave.” The amplitude is measured from the preceding negative trough as shown (*green double-headed arrow*).

In the present study, we sought to investigate the i-wave with respect to the myopia risk locus. First, we observed the effect on the i-wave in LA3 ERGs recorded in rare monogenic conditions that selectively impair ON, or both ON and OFF, pathway signaling. Second, we re-analyzed the LA3 ERGs previously recorded from TwinsUK participants, specifically to extract i-wave amplitudes (the prior study had only explored a-waves and b-waves in these responses). We hypothesized that these would associate with allelic identify at the risk locus given our earlier findings implicating the retinal OFF pathway.

## Methods

### Ethical Approval

The study had local research ethics committee approval (National Health Service Health Research Authority Research Ethics Service, Research Ethics Committee London–Harrow, Reference 11/LO/2029) and conformed to the tenets of the Declaration of Helsinki. Participants gave their written informed consent.

### Participants

The participants in this study were adult volunteers from the TwinsUK registry.^15^ They had been recruited to undergo ERG recordings as part of a study initially investigating heritability of retinal response parameters.^16^ Additional participants were patients, from specialist retinal clinics, with molecularly confirmed monogenic disorders affecting retinal signaling post-phototransduction. These were 10 patients with “congenital stationary night blindness” (CSNB), recruited for research recordings: six patients had complete CSNB (in whom ON bipolar cell signals are selectively lost); and four patients had incomplete CSNB (with impairment at the presynaptic terminal, affecting signal transmission to both ON and OFF bipolar cells).^17^^–^^19^

### ERG Recordings and Analyses

Participants underwent full-field dark-adapted and light-adapted ERG recordings compliant with the international standard.^20^ Conductive fiber electrodes (“DTL-plus” electrodes, Diagnosys LLC, Lowell, MA, USA) were placed in the lower conjunctival fornix; for seven of the patients, gold foil electrodes (CH Electrodes, UK) were used rather than conductive fiber electrodes. The pupils were pharmacologically dilated with 1.0% tropicamide and, in most cases, 2.5% phenylephrine. ERGs were recorded from both eyes. The specific waveforms analyzed in the present study were those elicited by the LA3 stimulus (a 3 cd m^−2^ white flash delivered on a 30 cd m^−2^ light-adapting white background, following at least 10 minutes of adaptation to the background). Stimulation and recording were with the Diagnosys Colordome system using Espion software (Diagnosys, Cambridge, UK). Typically, averages were taken from 60 flash presentations. Traces with high levels of noise or blink artifacts were excluded as described previously.^16^

Patient waveforms were qualitatively reviewed with specific focus on the presence or absence of the i-wave. ERGs from TwinsUK participants were analyzed with i-wave amplitudes measured. These were the ERGs that had been investigated in our previous study.^9^ However, in that study, only a-wave and b-wave parameters had been analyzed. In the present study, the i-wave was specifically investigated, with amplitude measurements made from the preceding trough (as depicted in Fig. 1). These measurements had not previously been made, and were undertaken independently by two investigators (authors J.K.T. and K.V.). In cases of disagreement, a third investigator, experienced in ERG analysis (author O.M.) arbitrated. No cases of disagreement (other than due to transcription errors) were found. All investigators were blind to participant genotype at the myopia risk locus when making the measurements.

### Autorefractions

Many TwinsUK participants had also undergone measurement of refractive error using an autorefractor (Humphrey-670, Humphrey Instruments, or ARM-10, Takagi Seiko). Spherical equivalent refractions were averaged from both eyes.

### Genetic Association Analyses

ERG i-wave amplitudes were averaged from both eyes of each TwinsUK participant when available. TwinsUK participant genotyping was done using Illumina HumanHap610Q chips and imputation was performed using Minimac3 software^21^ using haplotypes from Haplotype Reference Consortium r1.12016 as a reference.^22^ Association testing was performed using all genotyped subjects (for whom i-wave amplitudes were available), similar to our previously described methods.^9^ Testing was through a linear mixed model to assess genetic association with the electrophysiological parameter of interest (i-wave amplitude). In the linear mixed model-based method, population structure was included as fixed effects, whereas genetic kinship was modeled as the variance-covariance structure and used as a random effect. The model included the electrophysiological parameter as the outcome and allelic dosage at the rs524952 locus as an independent predictor, while simultaneously adjusting for age, sex, and intrafamilial relatedness, as implemented in the software GEMMA.^23^ Statistical significance was defined as an association with a *P* value less than 0.05. In a separate analysis, we additionally adjusted for refractive error where this was available.

(An analysis was also performed to explore the strength of association without adjusting for any covariates. Linear regression was performed without adjusting for effect of covariates using the software PLINK.)^24^

## Results

### Patient Recordings

**Figure 2.**
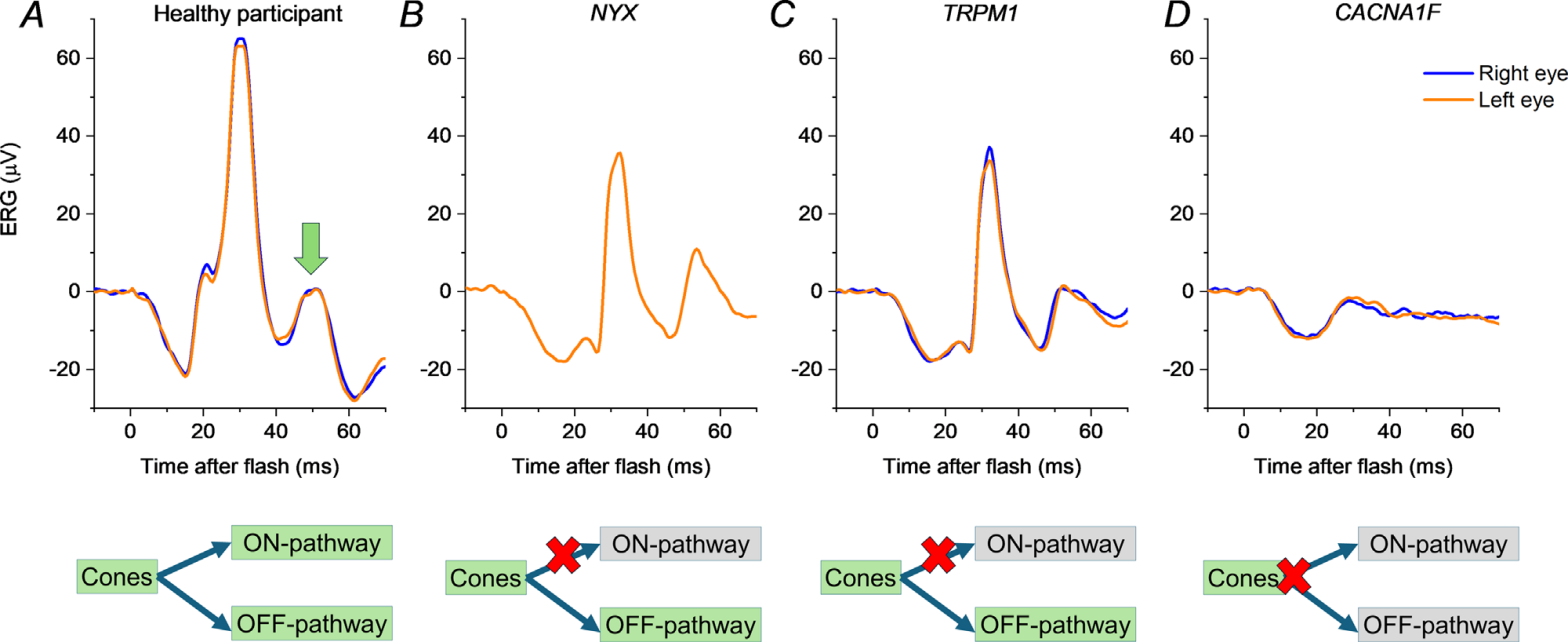
**LA3 ERGs in patients with post-phototransduction deficits, recorded with conductive fibre electrodes.** (**A**) Example ERGs from a healthy individual; the *green arrow* denotes the i-wave. (**B,** **C**) ERGs from patients with complete CSNB, entailing loss of ON pathway signals: **B** depicts recordings from a male patient hemizygous for a variant in *NYX* (c.106_111del p.(Ala36_Cys37del); reference sequence NM_022567.2); **C** depicts recordings from a female patient with biallelic variants in *TRPM1* (c.1780C>T p.(Arg594Trp) and c.2900G>A p.(Arg967Gln); and reference sequence NM_001252020.1). (**D**) ERGs from a male patient with incomplete CSNB (associated with a variant in *CACNA1F*, namely c.3213T>G p.(Asn1071Lys); reference sequence NM_005183.2), where both ON and OFF pathways are impaired. The i-wave appears preserved in the patients with complete CSNB **B** and **C**, but abolished in the patient with incomplete CSNB **D**.

**Figure 3.**
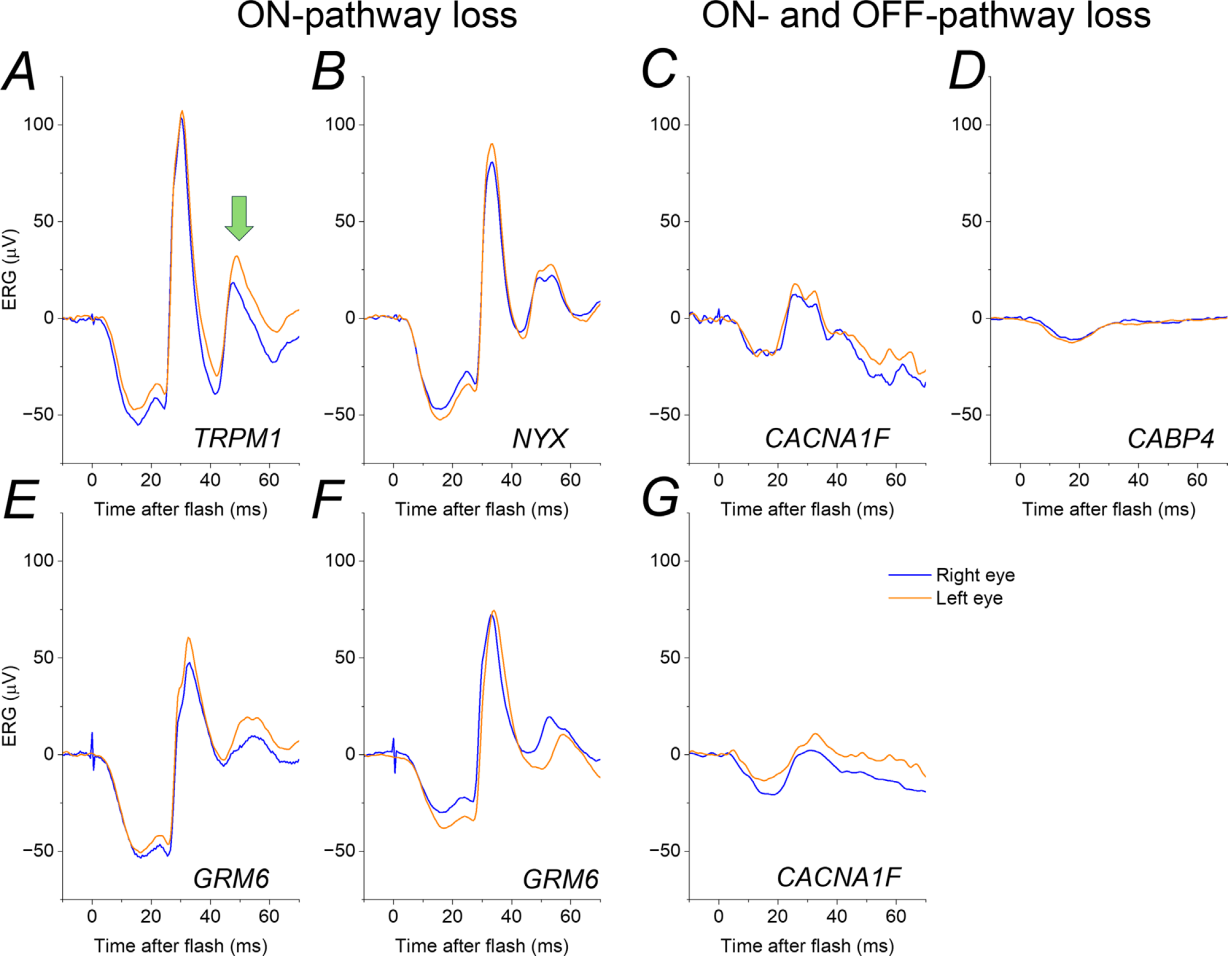
**LA3 ERGs in additional patients with complete and incomplete CSNB, recorded with gold foil electrodes.** Recordings from patients with genetic variants associated with complete CSNB (giving selective ON-pathway loss) are shown in (**A,** **B,** **E,** **F**). Recordings from patients with variants associated with incomplete CSNB (entailing attenuation of both ON- and OFF-pathways) are shown in (**C,** **D,** **G**). **A** Female homozygous for variant in *TRPM1* NM_001252020.1:c.2045T>C p.(Leu682Pro). **B** Male hemizygous for *NYX* NM_022567.2:c.647A>G p.(Asn216Ser). **C** Male hemizygous for *CACNA1F* NM_005183.2:c.4147G>A p.(Glu1383Lys). **D** Female homozygous for *CABP4* NM_145200.5:c.646C>T p.(Arg216*). **E** Female homozygous for *GRM6* NM_000843:C.19340G p.(Pro645Arg). **F** Female with biallelic variants in GRM6, c.58_72de1 p.(Trp20_Ala24del) and c.1957C>T p.(Arg653Cys); reference sequence, NM_000843.3. **G** Male hemizygous for *CACNA1F* NM_005183.2:c.784C>T p.(Arg262*). As in Figure 2, the i-wave (highlighted by the *green arrow* in **A**) persists in patients with selective ON-pathway loss and is not discernible in patients with both ON and OFF-signal attenuation.

Figure 2 shows ERGs from patients with genetically proven complete and incomplete CSNB, together with those of a healthy participant for comparison. In the patients with complete CSNB, the i-wave persists indicating that it does not arise in the retinal ON pathway. The i-wave is not discernible in the third patient, in whom both ON and OFF pathways are impaired. Figure 3 depicts similar data from 7 further patients in whom recordings were made using different electrodes. Taken together, these findings are consistent with an OFF pathway origin for the i-wave in the human LA3 ERG.

### TwinsUK Participant Recordings and Associations With Myopia Risk Locus

**Figure 4.**
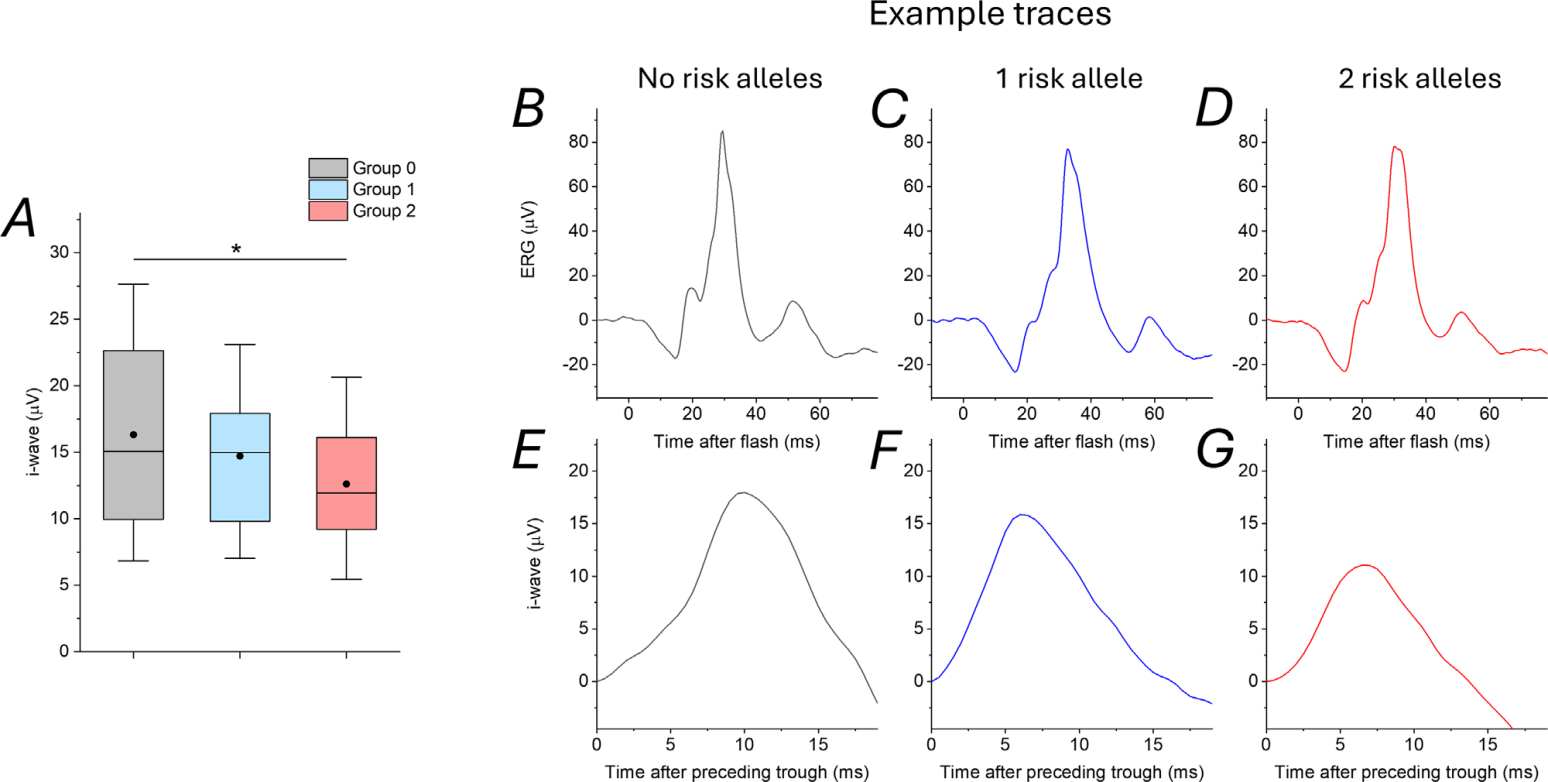
**Boxplots of i-wave amplitudes from participants with 0, 1,** **or 2 risk alleles (designated** **groups 0, 1,** **and 2,** **respectively) and example traces from each group.** (**A**) Boxplots. *Filled circles* show means. *Boxes* show medians and 25th and 75th centiles; and the *whiskers* show 10th and 90th centiles. The *asterisk* denotes significance for association between i-wave amplitude and genotype determined by linear mixed model, including all participants, with adjustment for age, sex and relatedness. (**B–D**) Example LA3 ERG traces (averaged from both eyes, approximately 60 flash presentations) from a representative participant from each group (examples chosen with i-wave amplitudes close to the mean/median of the respective group). (**E–G**) Expanded amplitude and time scales to better illustrate the i-wave (x-axis shows time after preceding trough).

Recordings and genotypes were available in 184 TwinsUK participants (95% female participants). Mean standard deviation (SD) age was 64.1 (SD = 9.7) years. Mean (SD) i-wave amplitude overall was 14.5 (SD = 6.5) microvolts. The Table groups participants by number of myopia-risk alleles at the locus of interest: 37, 101, and 46 participants had 0, 1, and 2 risk alleles, respectively; mean and median i-wave amplitudes are shown. Figure 4 presents box plots for the 3 groups as well as example traces from each group. Using the linear mixed model described in the Methods (with i-wave amplitude as outcome and allelic dosage as predictor, adjusting for age, sex, and familial relatedness), a significant association (*P* = 0.027) was found. (The *P* value obtained without adjusting for any covariates [see Methods] was 0.009.) Supplementary Table S1 gives these results in greater detail.

**Table.** Mean and Median I-Wave Amplitudes From Participants With 0, 1 or 2 Risk Alleles

			I-Wave Amplitude (Microvolts)	
Number of Risk Alleles	Number of Participants	Mean (SD) Age, Y	Mean (SD)	Median
0	37	63.1 (8.5)	16.3 (7.9)	15.1
1	101	64.6 (10.6)	14.7 (6.2)	15.0
2	46	63.7 (9.0)	12.6 (5.5)	12.0

### Comorbidities

The TwinsUK participants were mostly healthy, with over 90% having no documented ocular comorbidity. Four participants had glaucoma; three participants had age-related macular degeneration; four participants had diabetes (with none documented as having required retinopathy treatment); and one participant had previous treatment for retinal detachment in one eye. There was no significant difference found between allelic groups in prevalence of these comorbidities (both for each comorbidity individually or when grouped together).

### Adjustment for Refractive Error

Spherical equivalent refractive error was available for 175 participants. Mean (SD) spherical equivalent overall was −0.03 (SD = 2.38) diopters (D). The majority (87%) had a magnitude of refractive error less than 3 D. Only 4% had a spherical equivalent refractive error of greater magnitude than 6 D. When grouped by numbers of risk alleles, mean (SD) refractive errors were 0.36 (SD = 3.17), −0.03 (SD = 2.05), and −0.40 (SD = 2.28) D for participants with 0, 1, and 2 risk alleles, respectively. The difference in refractive error between groups was not significant (*P* > 0.22 for comparisons between groups, 2-tailed *t*-test). The association between allelic dosage and i-wave amplitude in these 175 individuals (linear mixed model as described, adjusting for age, sex, and familial relatedness) remained significant both before and after adjusting for refractive error (*P* = 0.035 and *P* = 0.043, respectively).

## Discussion

The present study explored the ERG i-wave as detected in the international standard light-adapted flash response (the LA3 ERG). Recordings in patients with rare genetically proven post-phototransduction deficits were consistent with an origin for this component residing in the cone-driven retinal OFF pathway. Subsequent investigation of ERGs recorded from genotyped adult participants from the TwinsUK cohort revealed a statistically significant association with a myopia-associated risk locus, adding further evidence that allelic identity at this locus is particularly associated with cone-driven OFF signals.

Prior reports have suggested the ERG i-wave originates in retinal OFF pathways,^11^^,^^12^ including pharmacological studies in nonhuman primates.^12^ In the present study, we investigated human LA3 ERGs, exploring responses in patients with complete CSNB (known to selectively impair ON bipolar cell signals) and incomplete CSNB (where dysfunction at the photoreceptor presynaptic terminal impairs transmission to both ON and OFF bipolar cells). The i-wave was present in the patients with complete CSNB; very similar findings were seen in these patients despite having distinct genetic causes. Inspection of LA3 responses from prior reports of complete CSNB also show that the i-wave is present, although this has not been explicitly noted. For example, Figure 1 of the study of Sergouniotis et al.^25^ shows clear presence of the i-wave in LA3 recordings from patients with *GRM6*-associated complete CSNB. In the present study, the patients with impairment of both ON and OFF signals had a more attenuated LA3 response with no discernible i-wave. Again, this is consistent with waveforms illustrated for incomplete CSNB in previous publications.^18^^,^^26^

**Figure 5.**
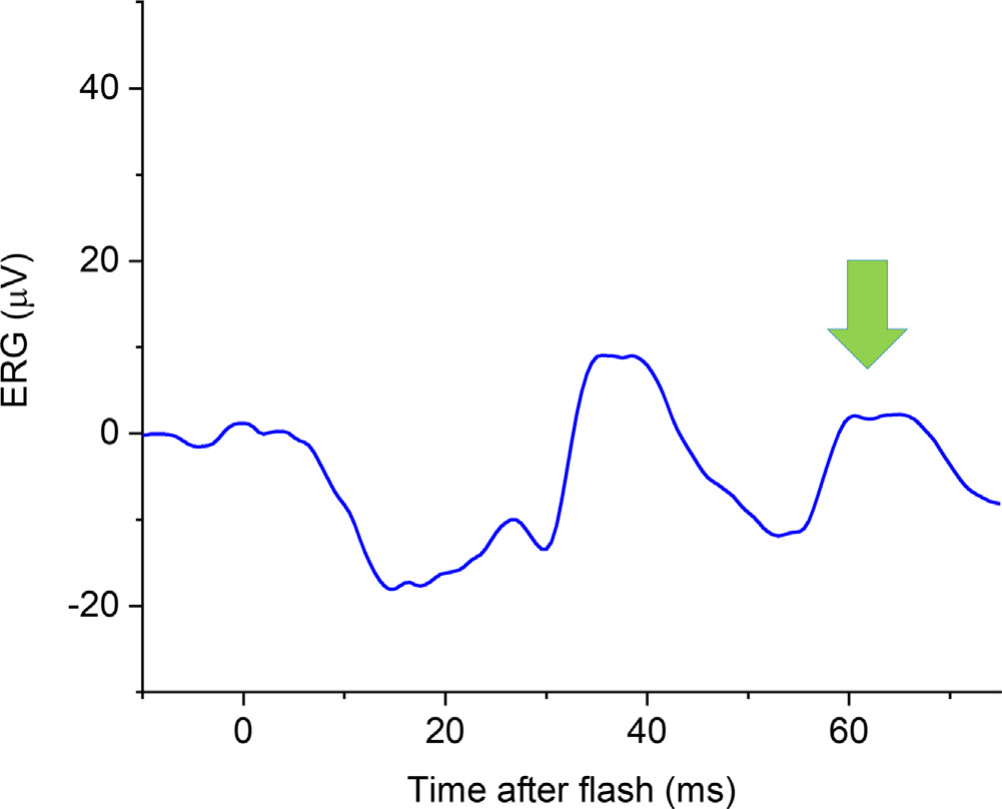
**Standard light-adapted single flash LA3 ERG recorded from a patient with melanoma-associated retinopathy.** The response shows similarity to that seen in patients with complete CSNB. The b-wave is attenuated, but the i-wave (highlighted by the *green arrow*) appears preserved.

Patients with melanoma-associated retinopathy can develop selective ON pathway dysfunction owing to development of autoantibodies to TRPM1.^27^^,^^28^ Thus, ERG findings are often similar to those seen in complete CSNB. Figure 5 shows the LA3 ERG recorded from a patient with melanoma-associated retinopathy, showing persistence of the i-wave. Again, re-assessment of waveforms in published reports also appear to show this phenomenon (for example, evident in the Supplementary Material of the case reported by Karatsai et al.^29^) Some patients with melanoma associated retinopathy can also develop concomitant OFF pathway impairment.^30^

Retinal ON and OFF pathway responses can be interrogated separately by delivering long duration flashes (lasting 150 to 200 ms), so that responses to stimulus onset and offset can be separated. This is an “extended protocol”^31^ endorsed by the International Society for the Clinical Electrophysiology of Vision and does not form part of the standard full-field ERG protocol.^20^ The findings of the present study suggest that specific attention to the presence of the i-wave in the standard LA3 ERG (for example, assessed relative to b-wave attenuation) could also give a clue to whether retinal ON pathways are selectively impaired; this could be of clinical utility particularly in patients who might not tolerate the extended protocol.

The second part of the present study quantified i-wave amplitudes from LA3 ERGs recorded from a largely healthy adult cohort. Based on our previous finding, in the same cohort, that cone-driven OFF signals appeared to be associated with the common myopia risk polymorphism,^9^ we hypothesized that a similar association would be seen with the i-wave. This was confirmed: a possible dose-response relation was evident (Fig. 4^5^) with, on average, lower i-wave amplitudes as the number of risk alleles increased. The association reached statistical significance, adjusting for age, sex, and familial relatedness.

ERG component amplitudes are correlated within the same individual and can be affected by factors such as electrode position.^32^ Given that we had previously detected an association between LA3 a-wave and b-wave amplitudes and the myopia risk locus, our present finding of association with another component of the same waveform might be expected. However, we then checked for associations between the myopia risk locus and other components within the same waveform (the troughs preceding and following the i-wave; see Fig. 1; which are part of the photopic negative response), and found no association. No significant association was found also between the locus and implicit times of any of these components (including i-wave peak time). In our previous study, no association was found with other ERG parameters, such as rod-driven ERG amplitudes or those components deriving mainly from the cone-driven ON pathway (such as the b-wave elicited by flashes delivered on a rod-saturating blue background), despite the electrode position being the same (all recordings performed within the same session). Taken together with the previous study, the results consistently point to an association between genotype at the myopia risk locus and components containing cone-driven OFF signals, rather than all ERG components.

ERG amplitudes can also vary with refractive error, with lower amplitudes seen in high myopes.^33^ Thus, it was theoretically possible that differences in refractive error could be confounding. However, a minority of participants had high refractive error, and there was no significant difference in mean refractive error between groups. Furthermore, even after adjusting for refractive error, the association between the risk locus and i-wave amplitudes remained significant. Thus, we conclude that the risk polymorphism is directly associated with retinal electrical signals (consistent with the proximity to the *GJD2* gene, which encodes retinal gap junctions), and that this makes a small contribution to the very large number of (genetic and environmental) factors that, in combination, determine whether an individual becomes myopic.

The association between this particular locus and refractive error has been consistently demonstrated in large GWAS analyses and meta-analyses that included tens of thousands of participants. That a significant association with refractive error was not seen in this study (184 individuals) is not unexpected. Refractive error is a complex trait arising from the combination of many, and as yet only partly identified, genetic and environmental factors, each conferring some (mostly small) risk. That we found a significant association between this locus and an ERG parameter in a relatively small number of individuals (compared with typical GWAS sizes) supports the hypothesis that genotype at this locus directly affects retinal electrophysiology (which in turn affects the risk of myopia) rather than the observed ERG associations being attributable simply to refractive error; this was further supported by the association persisting even after adjustment for refraction.

Our finding of specific association with cone-driven OFF signals does not exclude the possibility of associations between this locus and other retinal pathways that might emerge from larger studies with greater power or other types of functional study. Multiple different pathways are likely to be relevant, with the many genetic risk factors acting through numerous as yet undefined mechanisms. Many previous studies have demonstrated the importance of the ON-pathway in emmetropization. Humans with congenital loss of ON-pathway signals (complete CSNB) are usually highly myopic, although myopia is also a typical feature of *CACNA1F*-associated incomplete CSNB, in which there is attenuation of both ON and OFF pathways.^34^ Animal studies have yielded results that ostensibly point in different directions: ON-channel blockade in kitten eyes was shown to lead to hyperopia^35^; mice with *nyx* mutations (affecting ON-bipolar cell signals) were initially more hyperopic compared with wild-type mice, but showed greater susceptibility to form-deprivation myopia.^36^ Myopia is associated with choroidal thickness, and visual stimuli that stimulate ON or OFF pathways more strongly have been shown to affect choroidal thickness differently.^37^^,^^38^ In the general population, myopia is likely to arise from more subtle mechanisms relating to the balance between ON and OFF signals, potentially differing by retinal region, rather than selective profound loss of one particular pathway.

The largest meta-analysis to date, pooling data from over 540,000 individuals, has identified over 300 genetic loci associated with refractive error.^5^ Given the relatively small numbers in our cohort, we restricted our analysis to a hypothesis-driven investigation of one specific polymorphism (based on our prior findings and that this is a common polymorphism with one of the strongest myopia associations). Future ERG studies in significantly larger cohorts could potentially provide sufficient power to investigate the effects of many different genetic loci on retinal electrical signals.

Other limitations of our study, other than the relatively small sample size, include the specific demographics of our cohort (mostly White European ethnicity and female subjects). Future studies in cohorts with other demographics would be helpful. In addition, the mean age of our cohort was 64 years. In terms of relevance of our findings to myopia development, we are assuming that ERG recordings in adulthood will be reflective of retinal signals at earlier ages (because myopia usually develops earlier in life). Older age groups also have greater prevalence of comorbidities. However, despite the age range, the cohort was largely healthy: >90% had no known relevant comorbidity, and there were no significant differences between allelic groups.

In conclusion, our study has provided evidence consistent with the i-wave of the human LA3 ERG arising in retinal OFF pathways (hence providing a clinically useful marker to evaluate whether ON pathways have been selectively impaired). Second, we found an association between i-wave amplitudes and allelic dosage at a common myopia risk locus, strengthening further the evidence for a role for cone-driven signals in the pathogenesis of myopia.

## Supplementary Material

Supplement 1
