# Supplementary material for: The Electroretinogram I-Wave, a Component Originating in the Retinal OFF-Pathway, Associates With a Myopia Genetic Risk Polymorphism
Source: Invest Ophthalmol Vis Sci. 2024 Nov 12;65(13):21. doi: 10.1167/iovs.65.13.21 (PMC11562975; doi:10.1167/iovs.65.13.21)
Supplement: Supplement 1 [file iovs-65-13-21_s001.pdf]

Supplementary Table 1. Results of linear mixed model testing for association between genotype at the myopia risk locus (rs524952) and i-wave amplitudes before and after adjustment for covariates.

Participants	Number	Frequency of risk allele	Covariates	Beta (μV)	SE (μV)	p-value
All with genotypes and ERGs	184	0.524	None	-1.87	0.71	0.009
			Age, sex, familial relatedness	-1.83	0.82	0.027
All with genotypes, ERGs and refractions	175	0.511	Age, sex, familial relatedness	-1.80	0.85	0.035
			Age, sex, familial relatedness, spherical equivalent	-1.71	0.84	0.043
